# Reply to Pluta, R. Comment on “Minich et al. Is Melatonin the “Next Vitamin D”?: A Review of Emerging Science, Clinical Uses, Safety, and Dietary Supplements. *Nutrients* 2022, *14*, 3934”

**DOI:** 10.3390/nu15061507

**Published:** 2023-03-21

**Authors:** Deanna M. Minich, Melanie Henning, Catherine Darley, Mona Fahoum, Corey B. Schuler, James Frame

**Affiliations:** 1 Department of Human Nutrition and Functional Medicine, University of Western States, Portland, OR 97213, USA; 2 Department of Sports and Performance Psychology, University of the Rockies, Denver, CO 80202, USA; 3 College of Naturopathic Medicine, National University of Natural Medicine, Portland, OR 97201, USA; 4 School of Naturopathic Medicine, Bastyr University, Kenmore, WA 98028, USA; 5 School of Nutrition, Sonoran University of Health Sciences, Tempe, AZ 85282, USA; 6 Department of Online Education, Northeast College of Health Sciences, Seneca Falls, NY 13148, USA; 7 Natural Health International Pty., Ltd., Sydney, NSW 2000, Australia; 8 Symphony Natural Health, Inc., West Valley City, UT 84119, USA

We would like to thank Dr. Pluta for his thoughtful comments [1] on our review article [2] questioning whether melatonin is the “next vitamin D”. Specifically, Dr. Pluta detailed several aspects of melatonin’s functions in brain health and in neurodegenerative processes related to Alzheimer’s disease. From the research he presented, towards the end of these comments, he summarized: “*All these studies suggest that melatonin may be effective in preventing the pathology of amyloid and tau protein and modulating the metabolism of the amyloid protein precursor. I have doubts about the suggestion that melatonin is “next vitamin D” [sic]. Does vitamin D have the therapeutic properties described above for melatonin?*”

As we stated in our article, there are several similarities between melatonin and vitamin D: “Both act as hormones, affect multiple systems through their immune-modulating, anti-inflammatory functions, are found in the skin, and are responsive to sunlight and darkness. In fact, there may be similarities between the widespread concern about vitamin D deficiency as a “sunlight deficiency” and reduced melatonin secretion as a result of “darkness deficiency” from overexposure to artificial blue light”. In other words, the two have overlapping functions and complementary activities related to light exposure. Additionally, both compounds decline endogenously with age [3,4]. They are associated with increases in age-associated conditions such as cancer [5,6], cognitive impairment/dementia [7,8,9], autoimmune diseases [10,11,12], and even cardiovascular disease [13,14], thereby indicating that there may be an increased need state throughout the lifespan. Thus, we suggested that melatonin may even be a necessary “nutrient”, similar to vitamin D.

Our suggestion is not that one replaces the other, but that there may be some similarities in function. Furthermore, our intention in this review article was to provide a survey of multiple areas of clinical concern related to how melatonin and vitamin D may be involved in foundational mechanisms associated with a broad spectrum of disease pathologies, including their (1) anti-inflammatory activities [15]; (2) antioxidant potential [16]; and (3) mitochondrial regulation [17].

What Dr. Pluta seems to be asking is how vitamin D compares with melatonin in relation to amyloid and tau protein pathology. In his comment, he cited several studies indicating melatonin’s proficiency in accomplishing many functions related to amyloid metabolism and inhibiting the tau protein’s hyperphosphorylation. While these are important hallmarks of dementia, we would like to offer a fundamental mechanistic view of neurodegenerative diseases involving the three aforementioned mechanisms. 

Specifically, copious research has suggested that inflammation is part of the underlying dysfunction that can ultimately result in or exacerbate the dysregulation, aggregation, and lack of proper elimination of amyloid and tau proteins [18,19]. Moreover, oxidative stress in the brain has been associated with dementia [20,21,22,23], most likely due to the lipid-rich nature of the brain matter. As stated in our review paper, vitamin D and melatonin are fat-soluble antioxidants that can cross the blood–brain barrier. Finally, mitochondrial function is one of the lynchpins of healthy metabolism and neurological function. With damage to the mitochondria through free radical activity or inflammation, there will be impairments in energy production, which could have significant implications for brain health [24]. Mitochondrial dysfunction is becoming increasingly recognized as part of the more extensive pathology underlying neurodegeneration [25] and Alzheimer’s disease [26,27,28,29].

Separate from the established and emerging research aiming to support the shared mechanisms of vitamin D and melatonin in brain- and neurological-related disorders, there is some indication that vitamin D may play a role in amyloid [30,31,32,33,34] and tau protein [35,36,37] metabolism, which are the distinct areas that Dr. Pluta showed particular interest in, and concern about, regarding our provocative statement of query as to whether melatonin is the “next vitamin D”.

In conclusion, we are grateful to Dr. Pluta for raising the question about the similarities between vitamin D and melatonin and whether the two may be seen as having identical functions, particularly for brain and neurological health. To reiterate, while we would not want to suggest that the two are interchangeable, we documented that they could target common mechanistic pathways underlying the pathologies of several chronic diseases and even foster healthy brain aging.
